# The Effect of Amantadine on Agitation in the Pediatric Traumatic Brain Injury Population: A Case Series

**DOI:** 10.7759/cureus.42892

**Published:** 2023-08-03

**Authors:** Emily Hon, Abigail Case

**Affiliations:** 1 Department of Physical Medicine and Rehabilitation, Hospital of the University of Pennsylvania, Philadelphia, USA; 2 Division of Rehabilitative Medicine, Children’s Hospital of Philadelphia, Philadelphia, USA

**Keywords:** neurorehabilitation, pediatric tbi, traumatic brain injury, pediatrics rehabilitation, agitation, amantadine

## Abstract

In this report, we present a case series involving four pediatric patients who sustained a traumatic brain injury (TBI) and required intensive care unit admission immediately after the injury. In each of the four cases, amantadine was started during the acute care hospital admission to address agitation. Cases were retrieved from the electronic medical record at the Children’s Hospital of Philadelphia between July 1, 2020, and October 31, 2022. This case series describes clinical data on TBI presentation, amantadine administration, patient behavior, and hospital course relating to agitation. This is the first publication that reports the effect of amantadine on agitation in the acute phase of recovery in the pediatric TBI population. Improvement in agitation was observed within 48 hours of amantadine initiation in all four cases based on the primary team progress notes, as well as the quantity of pro re nata medications given for agitation. Resolution of agitation was also observed in all cases, though the time scale varied. No adverse events were reported in relation to amantadine use, supporting other reports that the medication may be well tolerated in the pediatric population. More research is needed to determine the optimal dose of amantadine for the pediatric population and whether amantadine hastens agitation resolution compared to the current standard of care.

## Introduction

Posttraumatic agitation after a traumatic brain injury (TBI) has been reported in up to 70% of patients in the acute care setting [1] and remains just as prevalent in the acute rehabilitation setting [2,3]. Agitation poses challenges to delivering medical care, including raising safety concerns for both the patient and caregiver, delaying treatment, increasing acute care admission length of stay, creating a barrier to achieving functional independence, and negatively impacting the discharge setting [1,3-6]. A systematic review published in 2019 found insufficient evidence to recommend any pharmacological intervention to address posttraumatic agitation [7]. Accordingly, there are currently no Food and Drug Administration (FDA)-approved medications for agitation following TBI. However, anecdotal evidence and expert opinion have favored the off-label use of beta-blockers, mood-regulating anticonvulsants, and antidepressants [8]. A second systematic review in 2019 suggested that a regimen of propranolol and an anti-epileptic agent may be beneficial in this population based on data from a small number of high-quality studies [9].

Amantadine has been studied for treating TBI patients who have disorders of consciousness. However, recent efforts have sought to elucidate its impact on posttraumatic agitation. Physiologically, amantadine acts as a N-methyl-D-aspartate (NMDA) receptor antagonist [10]. NMDA receptors are highly prevalent in neural plasticity pathways. Antagonism at these receptors can prevent harmful excessive excitation [10], suggesting that amantadine may serve a neuroprotective role in select circumstances. Additionally, amantadine increases dopaminergic neurotransmission [11]. Dopamine is known to both function in behavioral regulation and enhance arousal [11], suggesting potential benefits for use in agitated TBI patients. In particular, arousal enhancement may reduce agitation by allowing patients to communicate their needs more effectively, reduce confusion, and allow them to participate more meaningfully in therapies. Yet, evidence for amantadine’s effects on agitation in adult TBI patients has been inconsistent [7,12,13]. Furthermore, to date, published research about amantadine usage to treat agitation in pediatric TBI patients is nonexistent, although the medication itself is well tolerated by this population [14]. The present case series seeks to reduce this gap in knowledge by reporting the effect of amantadine on posttraumatic agitation in pediatric TBI patients.

Materials and methods

We describe the clinical courses of four pediatric TBI patients who received amantadine to treat agitation. Institutional Review Board approval was obtained on October 25, 2022. Patients were included in the retrospective chart review if they were hospitalized at the Children’s Hospital of Philadelphia for TBI between July 1, 2020, and October 31, 2022, were less than 18 years of age at the time of TBI, required intensive care unit (ICU) admission immediately after injury, and were started on amantadine during the acute care hospital admission. Further review of the medical record confirmed that the indication for amantadine initiation was specifically for agitation. Patients with a pre-existing history of brain injury and who used amantadine as a home medication were excluded. Four patients were identified that met the inclusion and exclusion criteria.

Medical charts were reviewed for data on TBI severity and mechanism, age at time of injury, duration of ICU admission, duration of acute care hospitalization, duration of acute rehabilitation admission (if applicable), the dose and indication of amantadine initiation, the duration of amantadine administration, and clinical data that reflected patient agitation. This included the number of days that agitation was reported in the primary provider’s notes, the quantity of pro re nata (PRN) medications that were administered for agitation, the number of days that physical restraints were ordered, and the requirement and duration of one-to-one observation by hospital staff.

## Case presentation

Table 1 provides a summary of patient demographics, TBI descriptions, and the duration of hospitalization, with hospital day zero being the day of presentation. The duration of hospitalization was further divided into time spent in the ICU and acute rehabilitation settings.

**Table 1:** Summary of patient demographics, TBI descriptions, and duration of hospital admissions. ICU: intensive care unit; GCS: Glasgow Coma Scale; TBI: traumatic brain injury

Patient	Gender	Age at the time of injury	Mechanism of injury	Brief description of initial imaging reports	Initial GCS in the hospital	Days in the ICU	Total days in acute care hospital	Days in acute rehab
1	Male	15	Bike and motor vehicle accident	Multifocal hemorrhagic contusions, scattered subarachnoid hemorrhage, small epidural hemorrhage	4	4	14	18
2	Male	14	Jet ski collision	Left-sided subdural hematoma and brain contusions, diffuse axonal injury	4	20	25	67
3	Male	15	Bike and motor vehicle accident	Hemorrhagic contusions to right occipital lobe and L inferior frontal lobe, subarachnoid hemorrhage to bilateral frontal lobes, right-sided subdural hemorrhage, diffuse axonal injury	14	5	12	7
4	Male	11	Penetrative injury from assault	Left temporoparietal intraparenchymal hematoma	13	9	18	81

Table 2 summarizes the data relating to amantadine administration, including when it was started in the hospital course, the total number of days the patient received amantadine, and the dose of amantadine the patient received during that period.

**Table 2:** Summary of patient amantadine use.

Patient	Hospital day of amantadine initiation	Dose of amantadine for agitation management	Days of amantadine use
1	10	50 mg daily	12
2	8	50 mg twice daily	250
3	5	50 mg twice daily	12
4	3	100 mg daily	41

Table 3 summarizes the indirect measures of agitation, such as the number of days agitation was documented in progress notes by the primary team, the quantity of PRN medication doses given for agitation, the number of days the patient had physical restraints in place, and the duration of one-to-one observation by hospital staff for any indication. Data from both pre- and post-amantadine initiation were included, if relevant.

**Table 3:** Summary of indirect measures of agitation, including data from both pre- and post-amantadine initiation.

	Number of days agitation documented in progress notes		Quantity of PRN medications given for agitation		Days in restraints		Duration of 1:1 observation	
Patient	Pre-amantadine	Post-amantadine	Pre-amantadine	Post-amantadine	Pre-amantadine	Post-amantadine	Pre-amantadine	Post-amantadine
1	9	0	1	0	4	0	6	7
2	6	14	0	0	6	12	0	22
3	7	1	0	0	7	1	1	7
4	2	2	1	0	2	9	1	19

Patient one

**Figure 1:**
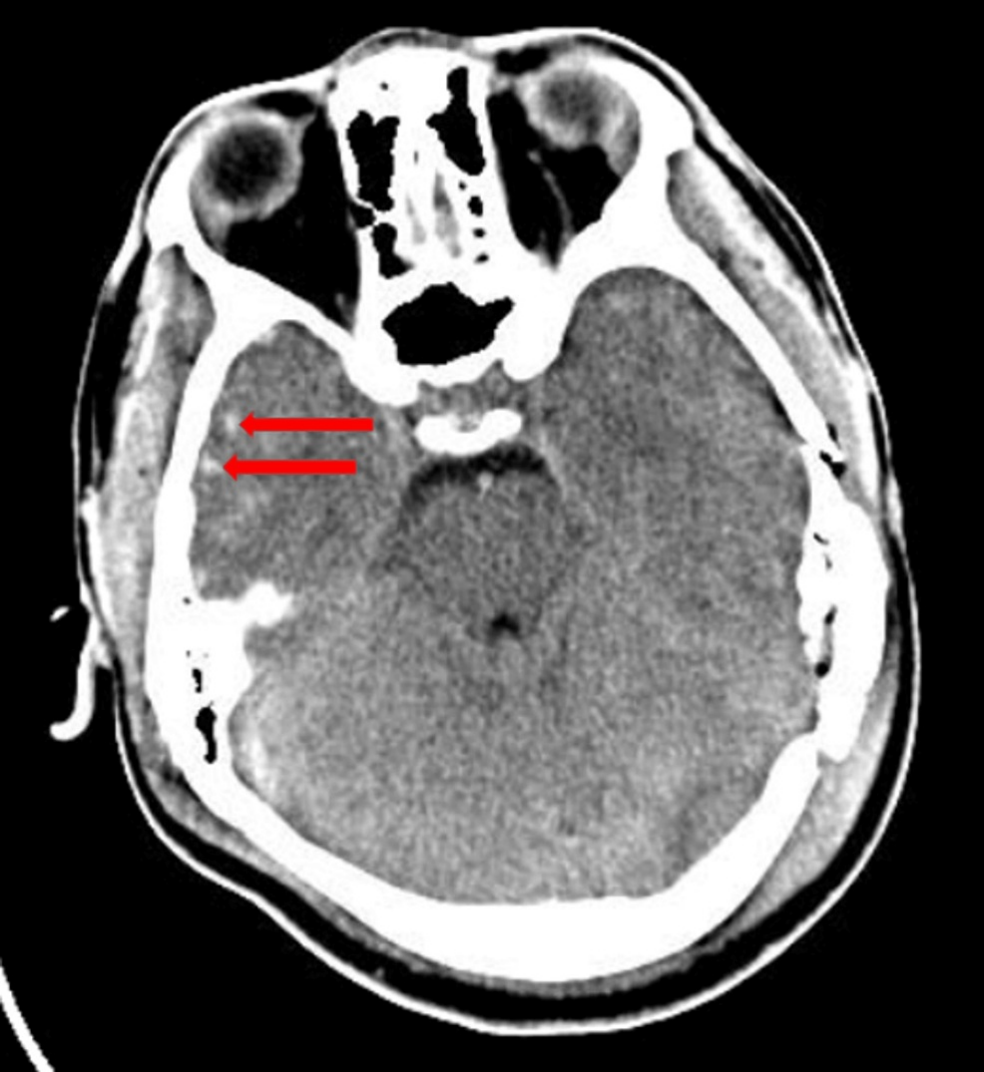
Computerized tomography of the head with increased areas of attenuation in the right temporal lobe consistent with hemorrhage contusions.

A 15-year-old male with no significant medical history presented to the hospital after he was struck by a motor vehicle at high speed while he was on his bicycle without a helmet. His head struck the windshield and then the ground. At the time of emergency medical services (EMS) arrival, he was conscious but confused. However, when he arrived at the hospital, his Glasgow Coma Scale (GCS) score was four. Specific eye, verbal, and motor (EVM) scores were not recorded. He was intubated for airway protection. Computerized tomography of the head (CTH) showed multifocal hemorrhagic contusions in the bilateral frontal and temporal lobes, scattered subarachnoid hemorrhages, and a small epidural hemorrhage at the right transverse and sigmoid sinus (Figure 1). Other imaging revealed multiple facial fractures, vertebral compression fractures, and a fracture of the left humerus. Neurosurgery did not recommend an acute surgical intervention. He was admitted to the ICU.

The patient was first noted to exhibit agitation on hospital day one (the day after the hospital presentation), where he paced, attempted to elope, pushed hospital staff, and tried to self-discontinue medical equipment multiple times. He required nonviolent restraints in the form of hand mitts for four days and one-to-one observation. He also received four doses of PRN lorazepam 1-2 mg to treat agitation. No other medications were trialed for agitation management. He spent four days in the ICU before he was transferred to the hospital floor, where he continued to have agitation.

On hospital day 10, the patient received his first dose of amantadine. He was started on amantadine 50 mg daily. Per primary team notes, the patient’s agitation resolved shortly after his first dose of amantadine was administered. He did not require any doses of PRN medications to address agitation thereafter. One-to-one observation was continued for seven more days after agitation resolved, for patient safety secondary to patient impulsivity. Amantadine was discontinued 12 days after it was initiated.

After 14 days at the acute care hospital, the patient was admitted to acute rehabilitation. He was discharged from the acute rehabilitation setting after 18 days. He returned home with his family. He did not experience any adverse events related to amantadine use.

Patient two

**Figure 2:**
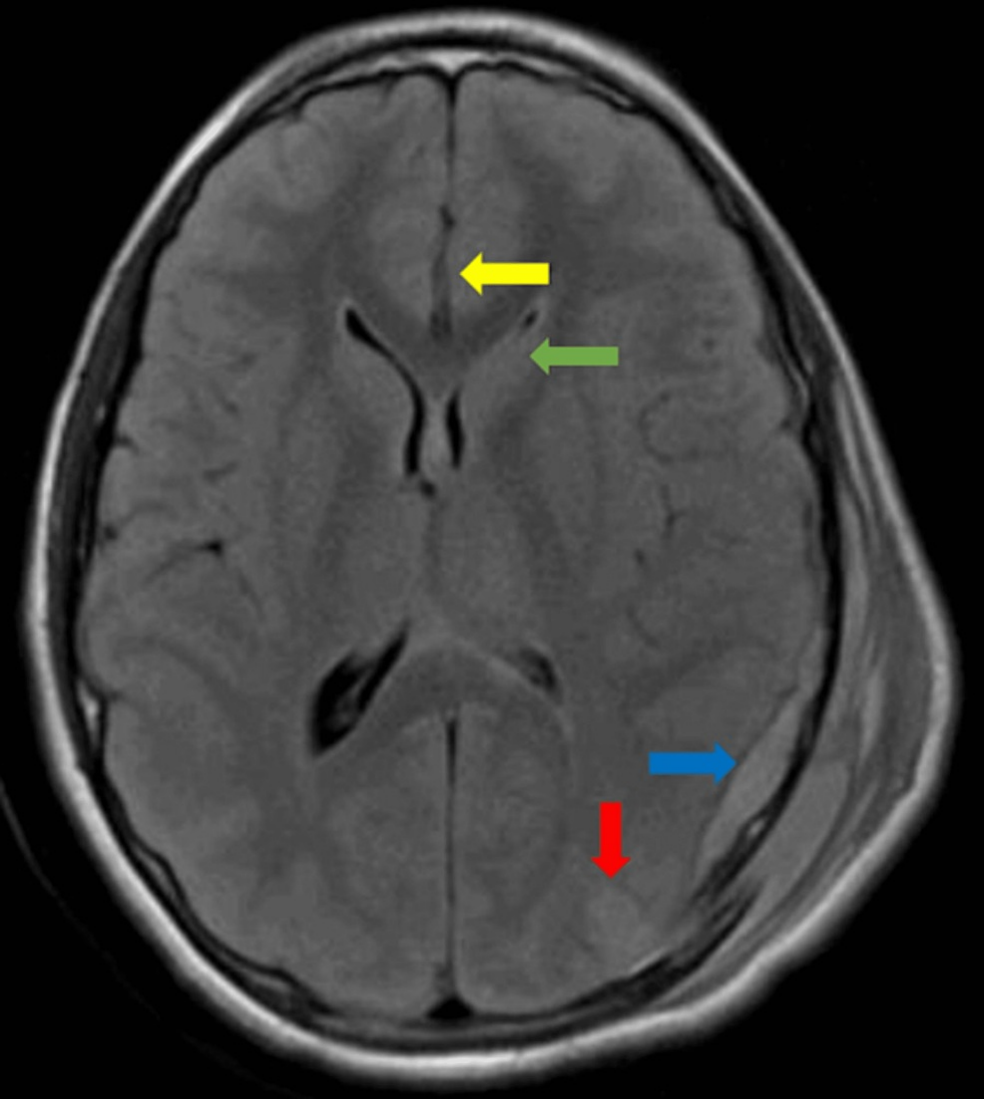
Magnetic resonance imaging of the brain with subdural hematoma (blue arrow), parenchymal hemorrhagic contusion (red arrow), mass effect on the frontal horn of the left lateral ventricle (green arrow), and rightward midline shift (yellow arrow).

A 14-year-old male with no significant medical history presented to the hospital after a jet ski collision where he was a passenger. When he was brought to land, he was unconscious, and bystanders began cardiopulmonary resuscitation. EMS arrived, return of spontaneous circulation was achieved, and he was intubated for airway protection. The initial GCS score at the hospital was four. Specific EVM scores were not recorded. A left-sided subdural hematoma with associated midline shift, brain contusions, multiple skull fractures, and diffuse axonal injury were identified on CTH and magnetic resonance imaging (MRI) of the brain (Figure 2). Other imaging was significant for rib fractures. An external ventricular drain was placed at the bedside for a significant and expanding intraventricular hemorrhage that ultimately required emergent left-sided craniotomy with epidural drain placement due to the elevation of previously depressed skull fractures and for evacuation of epidural and subdural hematomas. He was subsequently admitted to the ICU. His hospital course was complicated by paroxysmal sympathetic hyperactivity, which ultimately resolved with bromocriptine and propranolol, and persistent agitation.

The patient was first noted to have agitation on hospital day zero. On hospital day six, he failed extubation due to agitation and an ineffective trial of dexmedetomidine, requiring reintubation and sedation for safety. He was given multiple rescue doses of sedatives, including fentanyl and midazolam. He was then started on gabapentin for neuroirritability, without effect. On his eighth hospital day, he received his first dose of amantadine. He was titrated up to amantadine 50 mg twice daily. Bromocriptine was concurrently increased for the management of paroxysmal sympathetic hyperactivity. On hospital day 10, his agitation began to clinically improve. On hospital day 15, he was successfully extubated, and sedation was weaned to scheduled enteral morphine and lorazepam. He was then transferred to the hospital floor, where the patient’s agitation continued to improve.

The patient required nonviolent wrist restraints for a total of 18 days: six prior to amantadine initiation, and 12 after initiation. The one-to-one observation began the day after amantadine was initiated and remained in place for 22 days due to attempts to self-discontinue medical equipment and get out of bed without assistance. The patient’s agitation fully resolved 14 days after amantadine was initiated.

After 25 days at the acute care hospital, the patient was admitted to acute rehabilitation. During this admission, the patient’s suboptimal arousal was thought to be secondary to brain injury and was adversely affecting therapy sessions. The amantadine dose was titrated up to 100 mg twice daily to address this, as neurostimulation for arousal enhancement is an off-label use of amantadine. He was discharged from the acute rehabilitation setting after 67 days. He returned home with his family. He did not experience any adverse events related to amantadine use. He continued to receive amantadine in the outpatient setting until it was discontinued at a follow-up appointment; he received amantadine for a total of 250 days.

Patient three

**Figure 3:**
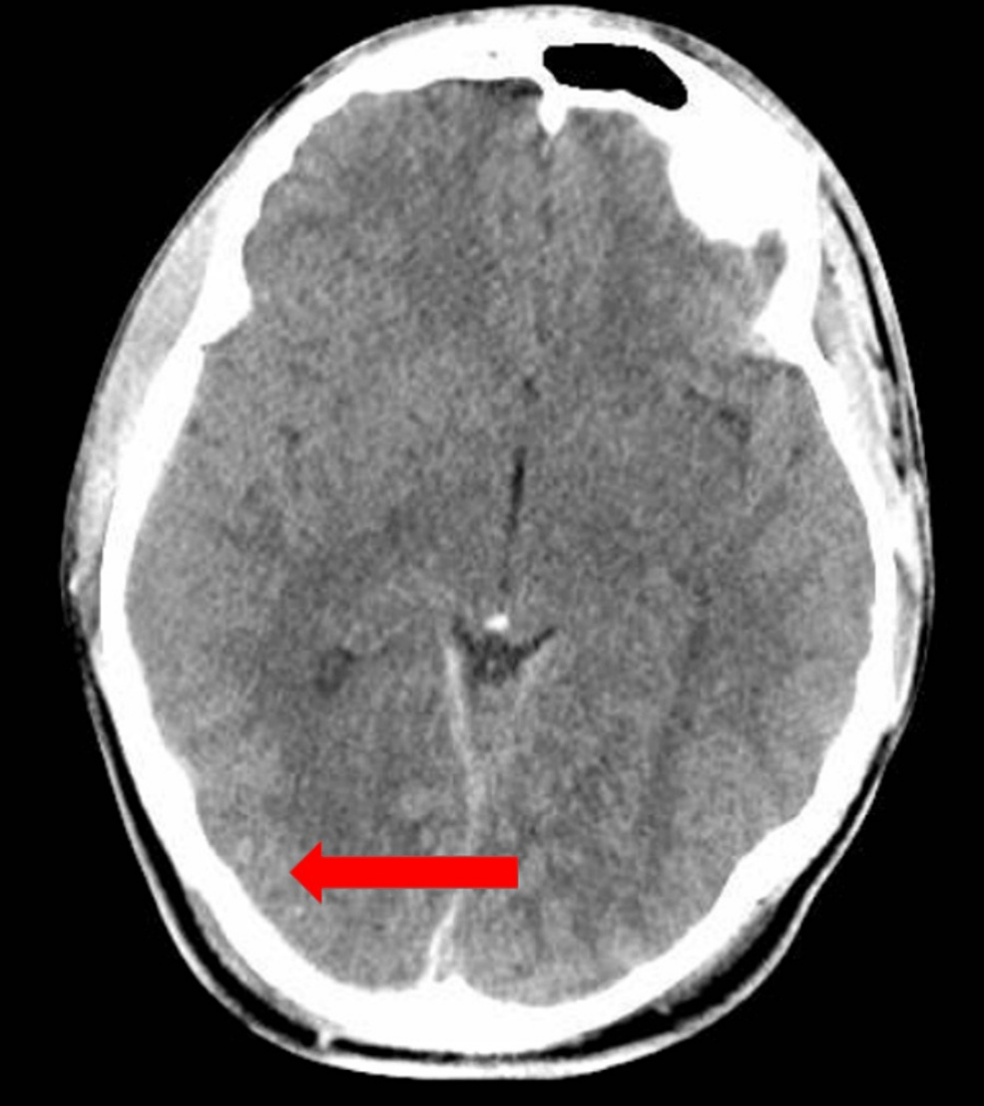
Computerized tomography of the head with intraparenchymal hemorrhagic contusions.

A 15-year-old male with no significant past medical history was brought to the hospital after he was struck by a motor vehicle while he was on his bicycle without a helmet. He was initially unconscious at the scene but regained consciousness. EMS brought the patient to the hospital, where the initial GCS score was 14. The eye-opening score was three, the verbal response was five, and the motor response was six. CTH showed hemorrhagic contusions to the right occipital lobe and left inferior frontal lobe, subarachnoid hemorrhages to bilateral frontal lobes, a right-sided subdural hemorrhage, and an occipital skull fracture (Figure 3). MRI brain additionally noted diffuse axonal injury. Other imaging was significant for a vertebral fracture, pulmonary contusions, and a right femur fracture. Neurosurgery did not recommend an acute surgical intervention, but he underwent open reduction and internal fixation of the right femur with the orthopedic surgery team. He was then admitted to the ICU. His ICU course was complicated by pain, which was managed with intravenous morphine 2 mg every three hours PRN and scheduled gabapentin 300 mg three times daily, which was titrated up from hospital days three through five. His course was additionally complicated by agitation.

The patient was first noted to exhibit agitation on hospital day one, as he was cursing at hospital staff, attempting to get out of bed without assistance, and trying to self-discontinue medical devices. Because of his agitation, he required a dexmedetomidine infusion that was quickly discontinued, received a one-time dose of intravenous lorazepam 1 mg, and required nonviolent wrist restraints. He later required one-to-one observation as well.

On his fifth hospital day, the patient was transferred to the hospital floor and received his first dose of amantadine. He was started on amantadine 50 mg twice daily. Subsequently, the patient’s agitation clinically resolved one day after amantadine was initiated. Restraints were discontinued at that time. He continued with one-to-one observation for impulsivity for an additional seven days.

After 12 days at the acute care hospital, the patient was admitted to acute rehabilitation. Amantadine was discontinued during this admission, 12 days after it was initiated. He was discharged from the acute rehabilitation setting after seven days. He returned home with his family. He did not experience any adverse events related to amantadine use.

Patient four

**Figure 4:**
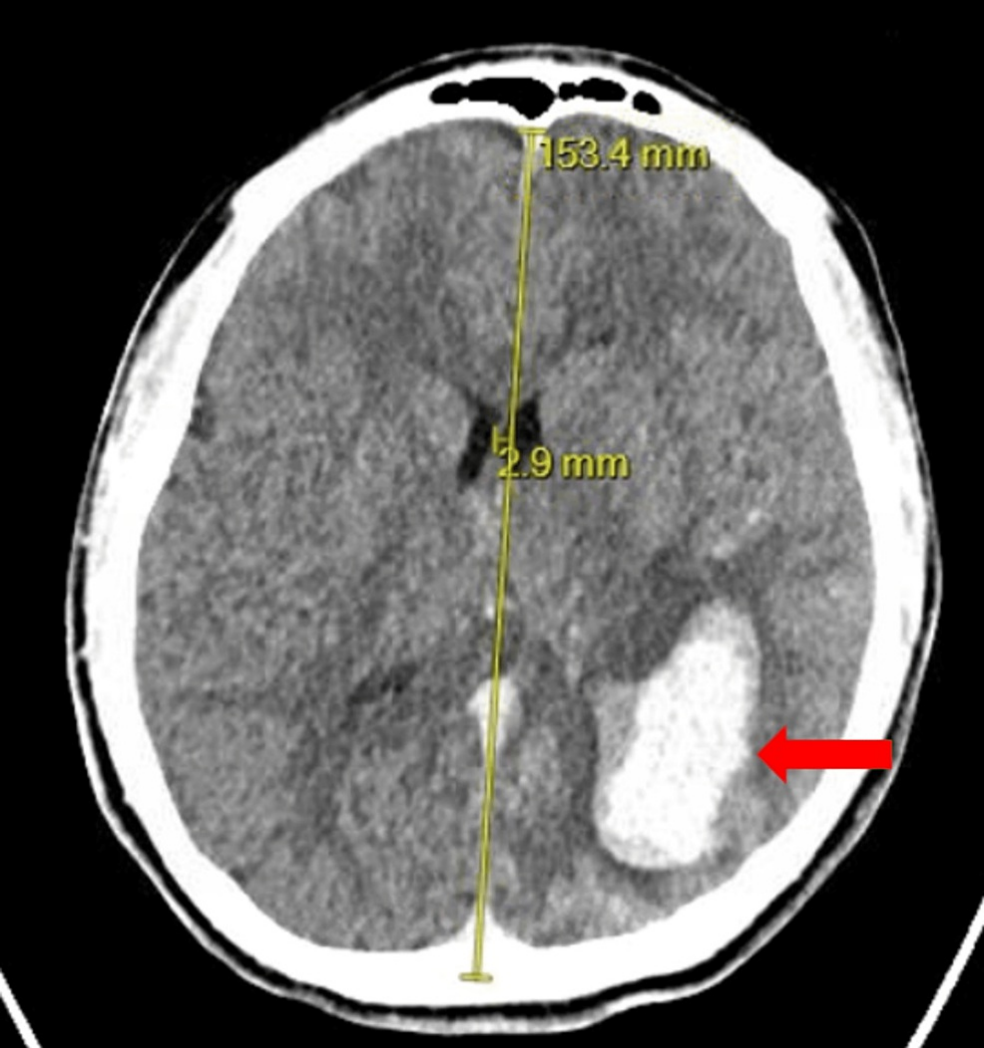
Computerized tomography of the head with left-sided temporoparietal intraparenchymal hemorrhage (red arrow) and 2.9 mm rightward midline shift.

An 11-year-old male with no significant past medical history was brought to the hospital after he was assaulted and sustained a non-gunshot wound penetrative injury to the head. The initial GCS score was 13. The eye-opening score was three, the verbal response was four, and the motor response was six. CTH was significant for a left temporoparietal intraparenchymal hemorrhage with midline shift and multiple skull fractures (Figure 4). His clinical examination acutely worsened, and he was taken emergently for craniotomy for hematoma evacuation and ligation of the arterial source with a surgical clip. He was transferred to the ICU postoperatively, where he was extubated without issue.

The patient was first noted to have agitation on hospital day one, as he was attempting to get out of bed without assistance and remove medical equipment. He was not ordered for any standing or PRN medication to manage agitation at this point. Subsequently, agitation worsened. He required one dose of PRN quetiapine 12.5 mg for agitation, nonviolent wrist restraints, and one-to-one observation for agitation management.

In an effort to manage ongoing agitation, the patient received his first dose of amantadine on his third day in the ICU. He was titrated up to amantadine 100 mg daily. The patient’s agitation clinically resolved two days after amantadine was initiated, and he did not require any additional PRN medications to treat agitation. However, wrist restraints remained in place for nine days and one-to-one observation remained in place for 19 days after amantadine was initiated; this was due to patient impulsivity, concern for patient safety, and protection of medical equipment. The only other medication change that occurred during the period of amantadine titration was an ongoing steroid taper, which was originally ordered to reduce intraparenchymal inflammation.

After 18 days at the acute care hospital, the patient was admitted to acute rehabilitation. Amantadine was discontinued during this admission, 41 days after it was initiated. He was discharged from the acute rehabilitation setting after 81 days. Of note, his rehabilitation discharge was repeatedly delayed due to nonmedical concerns. He ultimately returned home with his family. He did not experience any adverse events related to amantadine use.

## Discussion

To our knowledge, this is the first case series to describe the use of amantadine to treat agitation in the pediatric TBI population in the acute care setting. All four patients improved from an agitation standpoint after treatment with amantadine was initiated. Indeed, this was true for both descriptions in progress notes of primary team providers and the quantity of PRN medications used for agitation. Data on restraint use and one-to-one observation by hospital staff were recorded but correlated poorly as a measure for agitation, given common indications for both included protection of medical equipment and impulsivity, both of which were issues that these four patients contended with during their hospital courses. For three of the four cases, clinical resolution in agitation was demonstrated within 48 hours. In the final case of Patient two, the patient suffered severe TBI with a likely component of anoxic brain injury based on the pre-hospital course and continued to receive amantadine for 14 days before the resolution of clinically observed agitation was recorded. However, primary team progress notes indicated that agitation began to improve 48 hours after amantadine was initiated, suggesting some effect of the medication. However, in three of the four cases, other medication changes were made at the time of amantadine initiation to address other ongoing medical problems, including pain, paroxysmal sympathetic hyperactivity, and parenchymal inflammation. The extent that these other medication changes contributed to the improvement in agitation remains unclear. There were no adverse events related to amantadine use for any patient included in this study, supporting previous reports that amantadine may be well tolerated in the pediatric TBI population [14].

Although developing brains are intrinsically different from fully developed ones, past studies on the efficacy of amantadine on agitation in the adult TBI population will be discussed here, given the scarcity of corresponding literature in the pediatric population. Results have been mixed. On one hand, in 1988, a case series of two adult TBI patients reported swift resolution of persistent and refractory aggressive and agitated behaviors after amantadine was initiated [15]. More recently, two randomized, placebo-controlled studies that included 76 and 118 patients, respectively, assessed the effect of amantadine in adult TBI patients with irritability and aggression more than six months after injury, showing that symptoms significantly improved after treatment [16,17]. On the other hand, a retrospective cohort study of 139 patients was conducted in the trauma ICU and showed that the group that received amantadine was significantly more likely to have agitation [12]. Meanwhile, in a retrospective analysis of 12 brain injury patients, three patients were started on amantadine for severe agitation. Agitation completely resolved in two but amantadine had no discernable effect on the third [18].

The variability in the rate of improvement across the four patients in the present case series (as well as the past adult studies) may be due to several factors. First, the mechanisms of brain injuries, the brain structures that were affected, and the severity of TBI were heterogenous in our sample. It is possible that amantadine may act variably based on one or more of these factors. Second, the dosage of amantadine in a patient may be a factor in the person’s response. Because the range of children’s body weights can vary widely, giving the same daily dose of amantadine (in three of the four cases, a total dose of 100 mg in 24 hours was administered for agitation) can yield different results. There is a lack of consensus on an effective dose of amantadine in the pediatric population, not just to treat agitation but also for disorders of consciousness [19].

While this case series sheds light for the first time on the effectiveness of amantadine on agitation in pediatric TBI patients, it was limited by a small sample size. Additionally, all four patients discussed were male, and three of the four patients were teenagers, so it is unclear how generalizable these patients’ experiences would be for other demographic groups. Furthermore, because this study was retrospective in nature and dependent on the electronic medical record for data and accuracy, another limitation was that all data collected indirectly measured patient agitation. While direct measures of agitation, such as the Agitated Behavior Scale, do exist, they are not used consistently at this institution. As such, this study is unable to comment significantly on the severity of patient agitation. One measure that was additionally considered during the chart review was the quantity of standing and one-time orders of opioids, benzodiazepines, and antipsychotics. While it may have provided another indirect measure of agitation, ultimately the numerous indications for such medication use made this inclusion impractical.

Future studies would benefit from comparing pediatric TBI patients with agitation who received amantadine with those who did not. A controlled study would eliminate some of the challenges we encountered in this case series and may help tease out whether amantadine hastened the resolution of agitation or recovery in general, which has been suggested in previous case-controlled studies of the TBI population [14,20]. Next, a study that used direct measures of agitation would be helpful in identifying whether there is an optimal window of agitation severity to initiate amantadine use in this population. Additionally, several studies in the adult population on the efficacy of amantadine for chronic agitation have yielded promising results [16,17]. Our understanding of amantadine efficacy in the pediatric population would benefit from studying the effect of later initiation of amantadine in pediatric TBI patients with agitation. Finally, future studies should be done to determine the optimal dosing of amantadine for pediatric patients.

## Conclusions

This is the first study that reports on the effect of amantadine on agitation in the acute phase of recovery in the pediatric TBI population. Improvement in agitation was observed within 48 hours in all four cases, and full resolution of agitation was achieved in all cases, though the time scale varied. No adverse events were reported in relation to amantadine use, supporting that the medication may be well tolerated in the pediatric population. More research is needed to determine the optimal dose of amantadine for the pediatric population and whether amantadine hastens agitation resolution compared to the current standard of care.
